# Supplementary material for: Viral Long-Term Evolutionary Strategies Favor Stability over Proliferation
Source: Viruses. 2019 Jul 24;11(8):677. doi: 10.3390/v11080677 (PMC6722887; doi:10.3390/v11080677)
Supplement: Supplementary file 1 [file viruses-11-00677-s001.pdf]

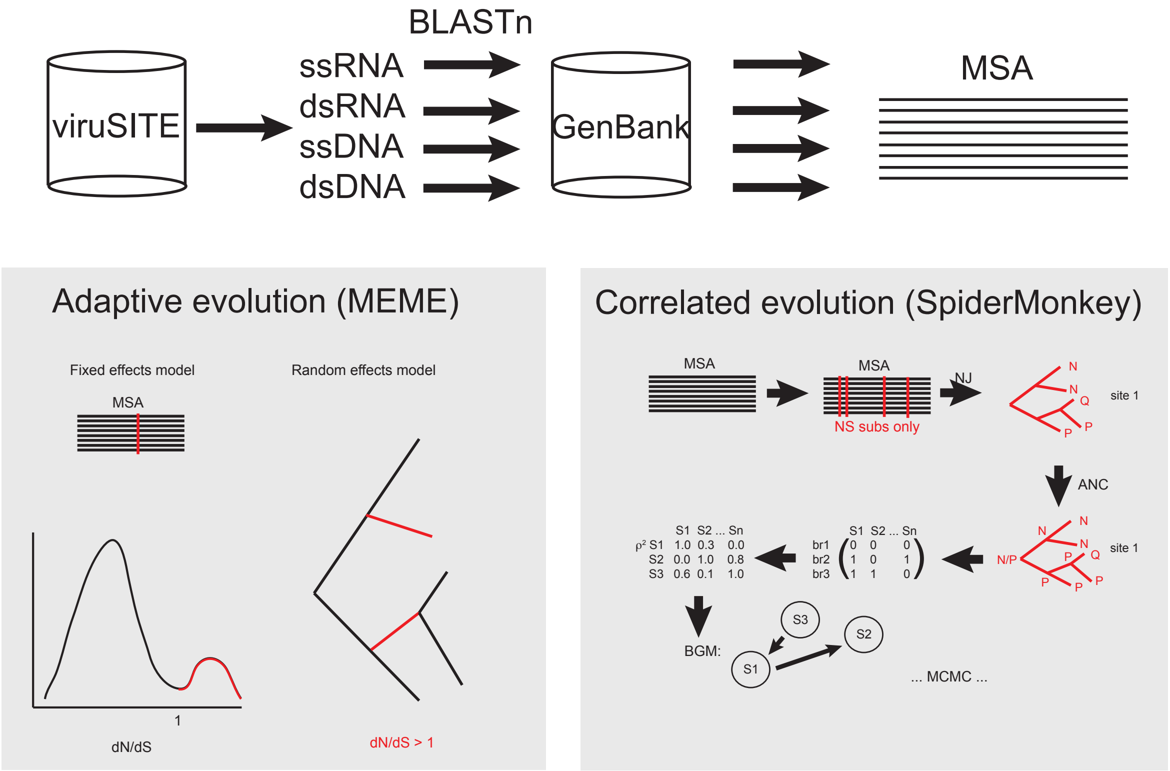

Figure S1: Overview of the experimental procedures. Nucleotide sequences of the four types of viruses (dsDNA, dsRNA, ssRNA, and ssDNA) were extracted from viruSITE, and stringent BLASTn searches were conducted on a custom GenBank NR database. These sequences were then aligned, and these multiple sequence alignments (MSA) were subjected to two independent sets of analyses: one searching for evidence for adaptive (diversifying) selection based on the MEME algorithm, and another one searching for evidence for correlated evolution based on the SpiderMonkey algorithm.

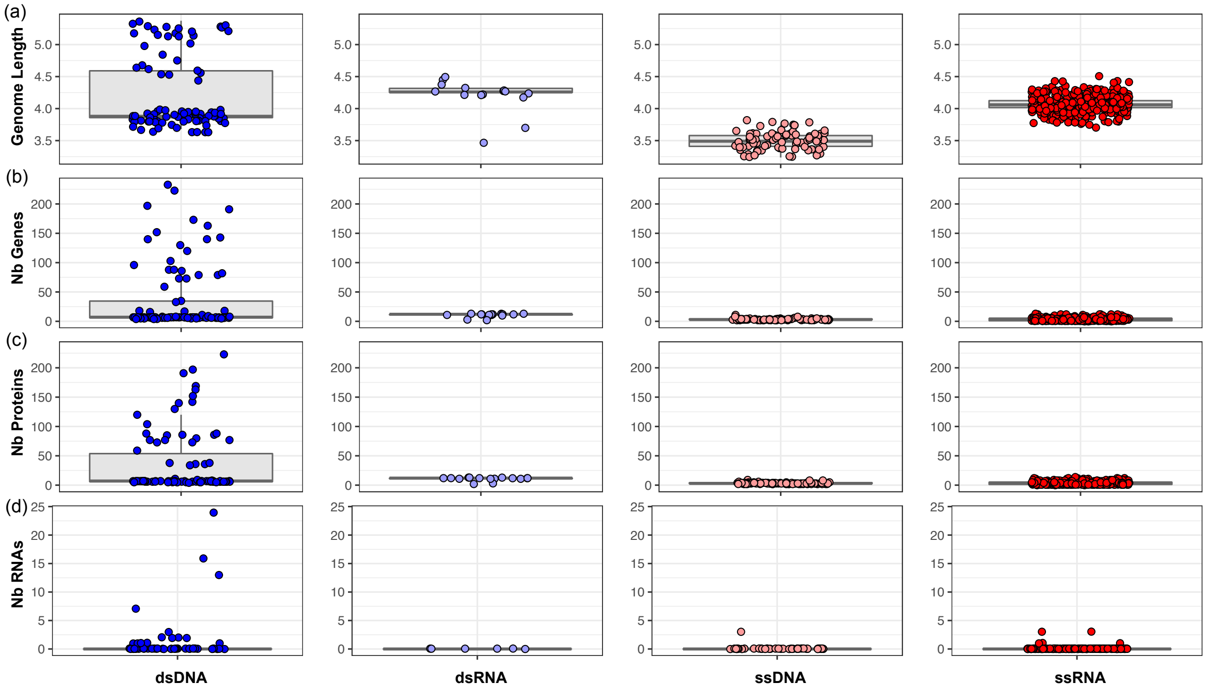

Figure S2: Physical characteristics of the four types of viruses. Boxplots show the distribution of four features: (a) genome size, on a $\log_{10}$ scale; (b) number of genes; (c) number of proteins; (d) number of RNAs. These distributions are shown for dsDNA (blue), dsRNA (purple), ssDNA (orange), and ssRNA (red).

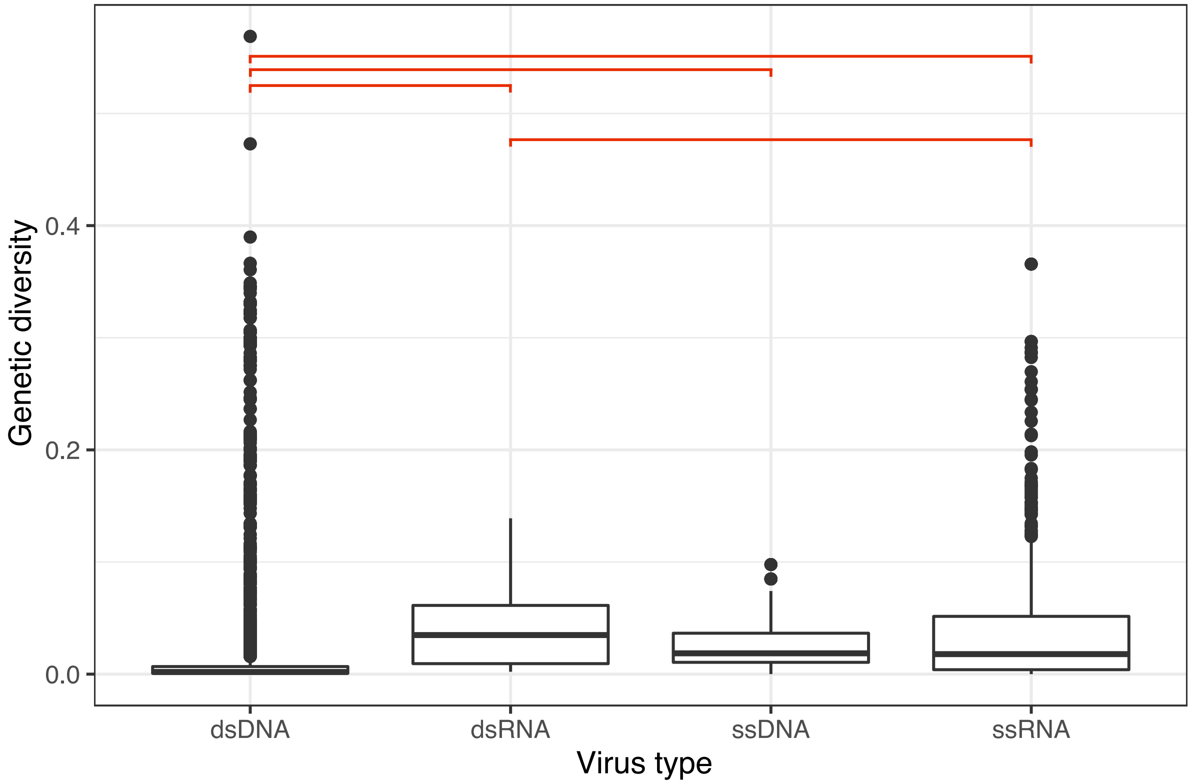

Figure S3: Viral diversity across viral types. Boxplots show the distribution of genetic diversity for each viral type, where diversity of each virus of each type is measured by computing the total tree length, divided by the number of sequences in each alignment. Similar results were obtained with mean patristic distances divided by number of sequences. Red bar indicate significant differences at the 1% false discovery rate level (Dunn test; Benjamini-Hochberg correction).

Table S1: GO enrichment tests for all the MEME genes of interests. Both tests are based on Fisher's exact test, either in its standard form ("classicFisher"), or in a version designed to be more conservative ("elimFisher"). $P < 0.01$ in bold. Broad viral stages (GO.ID column): E: cell entry/exit; R: replication; T: transcription; A: assembly.

VirTyp	GO	GO.ID	Term	Annotated	Significant	Expected	Rank in classicFisher	classicFisher	elimFisher
dsDNA	MF	**GO:0004003 (R)**	**ATP-dependent DNA helicase activity**	**122**	**70**	**20.94**	**1**	**1.80×10^{-26}**	**1.80×10^{-26}**
		**GO:0003677 (R)**	**DNA binding**	**343**	**107**	**58.86**	**9**	**4.30×10^{-14}**	**4.30×10^{-14}**
		**GO:0003724 (R)**	**RNA helicase activity**	**149**	**60**	**25.57**	**14**	**8.30×10^{-13}**	**8.30×10^{-13}**
		**GO:0005524**	**ATP binding**	**353**	**94**	**60.58**	**20**	**1.10×10^{-7}**	**1.10×10^{-7}**
		**GO:0003723 (R)**	**RNA binding**	**262**	**64**	**44.96**	**32**	**0.00056**	**0.00056**
		**GO:0003700 (T)**	**DNA binding transcription factor activit...**	**64**	**20**	**10.98**	**35**	**0.00338**	**0.00338**
		**GO:0019783 (R)**	**ubiquitin-like protein-specific protease...**	**5**	**4**	**0.86**	**36**	**0.00367**	**0.00367**
		**GO:0005515 (E)**	**protein binding**	**223**	**52**	**38.27**	**37**	**0.00635**	**0.00635**
		GO:0016773	phosphotransferase activity alcohol gro	32	11	5.49	39	0.01332	0.01332
		GO:0005506	iron ion binding	10	5	1.72	40	0.01711	0.01711
	BP	**GO:0006260 (R)**	**DNA replication**	**184**	**84**	**24.83**	**1**	**$<1\times10^{-30}$**	**5.30×10^{-30}**
		**GO:0019079 (R)**	**viral genome replication**	**216**	**71**	**29.15**	**3**	**1.00×10^{-16}**	**1.00×10^{-16}**
		**GO:0006275 (R)**	**regulation of DNA replication**	**25**	**11**	**3.37**	**28**	**0.00016**	**0.00016**
		**GO:0022900**	**electron transport chain**	**7**	**5**	**0.94**	**31**	**0.00071**	**0.00071**
		**GO:0040011 (E)**	**locomotion**	**14**	**7**	**1.89**	**32**	**0.00108**	**0.00108**
		**GO:1901566 (R)**	**organonitrogen compound biosynthetic pro...**	**37**	**11**	**4.99**	**34**	**0.00698**	**0.00698**
		**GO:0044283 (R)**	**small molecule biosynthetic process**	**7**	**4**	**0.94**	**35**	**0.00806**	**0.00806**
		**GO:0006383 (T)**	**transcription from RNA polymerase III pr...**	**4**	**3**	**0.54**	**36**	**0.00871**	**0.00871**
		GO:0002252	immune effector process	2	2	0.27	38	0.01811	0.01811
		GO:0009072	aromatic amino acid family metabolic pro...	2	2	0.27	39	0.01811	0.01811
	CC	**GO:0042025 (R)**	**host cell nucleus**	**66**	**20**	**6.66**	**1**	**1.50×10^{-6}**	**1.50×10^{-6}**
		**GO:0005666 (T)**	**DNA-directed RNA polymerase III complex**	**4**	**3**	**0.4**	**16**	**0.0037**	**0.0037**
		**GO:0005882 (R)**	**intermediate filament**	**4**	**3**	**0.4**	**17**	**0.0037**	**0.0037**
		GO:0033179	proton-transporting V-type ATPase V0 do	2	2	0.2	23	0.0101	0.0101
		GO:0045095	keratin filament	2	2	0.2	24	0.0101	0.0101
		GO:0046806	viral scaffold	2	2	0.2	25	0.0101	0.0101
		GO:0042597	periplasmic space	12	4	1.21	29	0.0255	0.0255
		GO:0005887	integral component of plasma membrane	3	2	0.3	31	0.0282	0.0282
		GO:0005929	cilium	3	2	0.3	32	0.0282	0.0282
		GO:0031226	intrinsic component of plasma membrane	3	2	0.3	33	0.0282	0.0282
ssRNA	MF	**GO:0003968 (R)**	**RNA-directed 5'-3' RNA polymerase activi...**	**257**	**84**	**28.5**	**1**	**3.90×10^{-27}**	**3.90×10^{-27}**
		**GO:0004197 (R)**	**cysteine-type endopeptidase activity**	**52**	**28**	**5.77**	**10**	**8.00×10^{-15}**	**8.00×10^{-15}**
		**GO:0004252 (R)**	**serine-type endopeptidase activity**	**79**	**29**	**8.76**	**18**	**4.90×10^{-10}**	**4.90×10^{-10}**
		**GO:0046983 (R)**	**protein dimerization activity**	**38**	**18**	**4.21**	**20**	**1.20×10^{-8}**	**1.20×10^{-8}**
		**GO:0046789 (E)**	**host cell surface receptor binding**	**31**	**16**	**3.44**	**21**	**1.70×10^{-8}**	**1.70×10^{-8}**
		**GO:0004482 (R)**	**mRNA (guanine-N7-)-methyltransferase act...**	**86**	**26**	**9.54**	**25**	**4.50×10^{-7}**	**4.50×10^{-7}**
		**GO:0005198 (R)**	**structural molecule activity**	**327**	**62**	**36.26**	**28**	**7.20×10^{-7}**	**7.20×10^{-7}**
		**GO:0003725 (R)**	**double-stranded RNA binding**	**28**	**13**	**3.1**	**30**	**2.00×10^{-6}**	**2.00×10^{-6}**
		**GO:0004483 (R)**	**mRNA (nucleoside-2'-O-)-methyltransferas...**	**27**	**12**	**2.99**	**32**	**9.10×10^{-6}**	**9.10×10^{-6}**
		**GO:0008168 (R)**	**methyltransferase activity**	**113**	**37**	**12.53**	**16**	**6.30×10^{-11}**	**1.90×10^{-5}**
	BP	**GO:0006508 (R)**	**proteolysis**	**120**	**49**	**15.71**	**2**	**4.40×10^{-16}**	**4.40×10^{-16}**
		**GO:0032259 (R)**	**methylation**	**66**	**29**	**8.64**	**11**	**1.60×10^{-10}**	**1.60×10^{-10}**
		**GO:0019087 (R)**	**transformation of host cell by virus**	**43**	**21**	**5.63**	**14**	**6.90×10^{-9}**	**6.90×10^{-9}**
		**GO:0019082 (R)**	**viral protein processing**	**20**	**13**	**2.62**	**16**	**7.20×10^{-8}**	**7.20×10^{-8}**
		**GO:0019058**	**viral life cycle**	**411**	**87**	**53.81**	**13**	**6.70×10^{-9}**	**1.20×10^{-5}**
		**GO:0016070**	**RNA metabolic process**	**413**	**76**	**54.07**	**17**	**8.90×10^{-5}**	**8.90×10^{-5}**
		**GO:0019064 (E)**	**fusion of virus membrane with host plasm...**	**48**	**16**	**6.28**	**18**	**0.00019**	**0.00019**
		**GO:0019048**	**modulation by virus of host morphology o...**	**84**	**39**	**11**	**3**	**5.10×10^{-15}**	**0.00021**
		**GO:0030683**	**evasion or tolerance by virus of host im...**	**24**	**10**	**3.14**	**29**	**0.00043**	**0.00043**
		**GO:0039694 (R)**	**viral RNA genome replication**	**13**	**7**	**1.7**	**34**	**0.0005**	**0.0005**
	CC	**GO:0019031**	**viral envelope**	**164**	**51**	**25.27**	**2**	**9.60×10^{-9}**	**9.60×10^{-9}**
		**GO:0019012**	**virion**	**601**	**128**	**92.62**	**1**	**3.20×10^{-14}**	**3.00×10^{-8}**
		**GO:0055036**	**virion membrane**	**24**	**11**	**3.7**	**5**	**0.00033**	**0.00033**
		GO:0044454	nuclear chromosome part	11	5	1.7	6	0.01712	0.01712
		GO:0000778	condensed nuclear chromosome kinetochore	10	4	1.54	7	0.05346	0.05346
		GO:0000780	condensed nuclear chromosome centromeri	10	4	1.54	8	0.05346	0.05346
		GO:0000942	condensed nuclear chromosome outer kinet...	10	4	1.54	9	0.05346	0.05346
		GO:0042729	DASH complex	10	4	1.54	10	0.05346	0.05346
		GO:0072686	mitotic spindle	10	4	1.54	11	0.05346	0.05346
		GO:0019030	icosahedral viral capsid	3	2	0.46	12	0.06362	0.06362

Table S2: GO enrichment tests for all the SM genes of interests. Both tests are based on Fisher's exact test, either in its standard form ("classicFisher"), or in a version designed to be more conservative ("elimFisher"). $P < 0.01$ in bold. Broad viral stages (GO.ID column): E: cell entry/exit; R: replication; T: transcription; A: assembly.

VirTyp	GO	GO.ID	Term	Annotated	Significant	Expected	Rank in classicFisher	classicFisher	elimFisher
dsDNA	MF	**GO:0004003 (R)**	**ATP-dependent DNA helicase activity**	**122**	**51**	**28.17**	**1**	**1.10×10^{-6}**	**1.10×10^{-6}**
		**GO:0003677 (R)**	**DNA binding**	**343**	**109**	**79.21**	**4**	**1.30×10^{-5}**	**1.30×10^{-5}**
		**GO:0050662 (R)**	**coenzyme binding**	**10**	**8**	**2.31**	**8**	**0.00022**	**0.00022**
		**GO:0016616 (E)**	**oxidoreductase activity acting on the C**	**4**	**4**	**0.92**	**9**	**0.0028**	**0.0028**
		**GO:0016984**	**ribulose-bisphosphate carboxylase activi...**	**6**	**5**	**1.39**	**10**	**0.00312**	**0.00312**
		**GO:0004484 (T)**	**mRNA guanylyltransferase activity**	**11**	**7**	**2.54**	**14**	**0.00456**	**0.00456**
		**GO:0020037 (E)**	**heme binding**	**11**	**7**	**2.54**	**15**	**0.00456**	**0.00456**
		**GO:0016773 (R)**	**phosphotransferase activity alcohol gro**	**32**	**14**	**7.39**	**21**	**0.00715**	**0.00715**
		**GO:0031423**	**hexon binding**	**7**	**5**	**1.62**	**23**	**0.00885**	**0.00885**
		GO:0019783	ubiquitin-like protein-specific protease...	5	4	1.15	24	0.01145	0.01145
	BP	**GO:0006260 (R)**	**DNA replication**	**184**	**59**	**40.49**	**2**	**0.00039**	**0.00039**
		**GO:0015977 (R)**	**carbon fixation**	**6**	**5**	**1.32**	**3**	**0.00247**	**0.00247**
		**GO:0022900**	**electron transport chain**	**7**	**5**	**1.54**	**5**	**0.00708**	**0.00708**
		**GO:0044283 (R)**	**small molecule biosynthetic process**	**7**	**5**	**1.54**	**6**	**0.00708**	**0.00708**
		**GO:1901566 (R)**	**organonitrogen compound biosynthetic pro...**	**37**	**15**	**8.14**	**7**	**0.00771**	**0.00771**
		**GO:0007166**	**cell surface receptor signaling pathway**	**5**	**4**	**1.1**	**9**	**0.00952**	**0.00952**
		GO:0007276	gamete generation	10	6	2.2	10	0.01012	0.01012
		GO:0019953	sexual reproduction	10	6	2.2	11	0.01012	0.01012
		GO:0032504	multicellular organism reproduction	10	6	2.2	12	0.01012	0.01012
		GO:0044703	multi-organism reproductive process	10	6	2.2	13	0.01012	0.01012
	CC	**GO:0042597 (E)**	**periplasmic space**	**12**	**7**	**1.98**	**5**	**0.00112**	**0.0011**
		**GO:0043229**	**intracellular organelle**	**101**	**27**	**16.68**	**7**	**0.00389**	**0.0039**
		**GO:0032991**	**macromolecular complex**	**96**	**28**	**15.86**	**3**	**0.00072**	**0.004**
		**GO:0030089**	**phycobilisome**	**3**	**3**	**0.5**	**8**	**0.00443**	**0.0044**
		**GO:0031514 (E)**	**motile cilium**	**3**	**3**	**0.5**	**9**	**0.00443**	**0.0044**
		GO:0019033	viral tegument	10	5	1.65	16	0.01436	0.0144
		GO:0000428	DNA-directed RNA polymerase complex	4	3	0.66	17	0.01557	0.0156
		GO:0005666	DNA-directed RNA polymerase III complex	4	3	0.66	18	0.01557	0.0156
		GO:0030880	RNA polymerase complex	4	3	0.66	19	0.01557	0.0156
		GO:0055029	nuclear DNA-directed RNA polymerase comp...	4	3	0.66	20	0.01557	0.0156
ssRNA	MF	**GO:0046789 (E)**	**host cell surface receptor binding**	**31**	**7**	**1.07**	**1**	**5.40×10^{-5}**	**5.40×10^{-5}**
		**GO:0003950 (T)**	**NAD+ ADP-ribosyltransferase activity**	**5**	**3**	**0.17**	**3**	**0.00037**	**0.00037**
		**GO:0005198 (R)**	**structural molecule activity**	**327**	**20**	**11.32**	**7**	**0.00369**	**0.00369**
		**GO:0016798 (E)**	**hydrolase activity acting on glycosyl b**	**12**	**3**	**0.42**	**8**	**0.00688**	**0.00688**
		**GO:0004482 (T)**	**mRNA (guanine-N7-)-methyltransferase act...**	**86**	**8**	**2.98**	**9**	**0.00772**	**0.00772**
		GO:0004308	exo-alpha-sialidase activity	6	2	0.21	13	0.0161	0.0161
		GO:0016997	alpha-sialidase activity	6	2	0.21	14	0.0161	0.0161
		GO:0003968	RNA-directed 5'-3' RNA polymerase activi...	257	15	8.89	16	0.02182	0.02182
		GO:0004553	hydrolase activity hydrolyzing O-glycos	10	2	0.35	19	0.04422	0.04422
		GO:0000702	oxidized base lesion DNA N-glycosylase a...	2	1	0.07	20	0.06804	0.06804
	BP	**GO:0006471 (T)**	**protein ADP-ribosylation**	**5**	**3**	**0.18**	**1**	**0.00039**	**0.00039**
		**GO:0006352 (T)**	**DNA-templated transcription initiation**	**4**	**2**	**0.14**	**8**	**0.007**	**0.007**
		**GO:0055085 (E)**	**transmembrane transport**	**23**	**4**	**0.81**	**9**	**0.00736**	**0.00736**
		GO:0006370	7-methylguanosine mRNA capping	63	6	2.23	11	0.02051	0.02051
		GO:0009452	7-methylguanosine RNA capping	63	6	2.23	12	0.02051	0.02051
		GO:0036260	RNA capping	63	6	2.23	13	0.02051	0.02051
		GO:0019068	virion assembly	66	6	2.33	14	0.02532	0.02532
		GO:0006397	mRNA processing	68	6	2.4	15	0.02891	0.02891
		GO:0016071	mRNA metabolic process	79	6	2.79	16	0.0548	0.0548
		GO:0005975	carbohydrate metabolic process	12	2	0.42	17	0.06435	0.06435
	CC	**GO:0019031 (E)**	**viral envelope**	**164**	**19**	**9.82**	**3**	**0.0016**	**0.0016**
		**GO:0019013**	**viral nucleocapsid**	**104**	**14**	**6.23**	**5**	**0.0019**	**0.0019**
		GO:0019012	virion	601	49	35.98	1	2.40×10^{-5}	0.015
		GO:0055036	virion membrane	24	4	1.44	6	0.0498	0.0498
		GO:0009654	photosystem II oxygen evolving complex	1	1	0.06	7	0.0599	0.0599
		GO:0019898	extrinsic component of membrane	1	1	0.06	8	0.0599	0.0599
		GO:1990204	oxidoreductase complex	1	1	0.06	9	0.0599	0.0599
		GO:0033180	proton-transporting V-type ATPase V1 do	2	1	0.12	10	0.1162	0.1162
		GO:0033644	host cell membrane	12	2	0.72	11	0.1582	0.1582
		GO:0009521	photosystem	3	1	0.18	12	0.1692	0.1692

Table S3: GO enrichment tests for all the SM+MEME genes of interests. Both tests are based on Fisher's exact test, either in its standard form ("classicFisher"), or in a version designed to be more conservative ("elimFisher"). $P < 0.01$ in bold. Broad viral stages (GO.ID column): E: cell entry/exit; R: replication; T: transcription; A: assembly.

VirTyp	GO	GO.ID	Term	Annotated	Significant	Expected	Rank in classicFisher	classicFisher	elimFisher
dsDNA	MF	**GO:0005198 (R)**	**structural molecule activity**	**327**	**9**	**2.54**	**1**	**6.00×10^{-5}**	**6.00×10^{-5}**
		GO:0004252	serine-type endopeptidase activity	79	2	0.61	2	0.12	0.12
		GO:0008236	serine-type peptidase activity	90	2	0.7	3	0.15	0.15
		GO:0017171	serine hydrolase activity	90	2	0.7	4	0.15	0.15
		GO:0004175	endopeptidase activity	133	2	1.03	5	0.28	0.28
		GO:0070011	peptidase activity acting on L-amino ac	149	2	1.16	6	0.32	0.32
		GO:0008233	peptidase activity	153	2	1.19	7	0.34	0.34
		GO:0140096	catalytic activity acting on a protein	191	2	1.48	8	0.45	0.45
		GO:0003677	DNA binding	343	2	2.66	9	0.79	0.79
		GO:0016787	hydrolase activity	402	2	3.12	10	0.86	0.86
	BP	**GO:0043158**	**heterocyst differentiation**	**6**	**2**	**0.02**	**1**	**0.00012**	**0.00012**
		**GO:0051726**	**regulation of cell cycle**	**7**	**2**	**0.02**	**2**	**0.00016**	**0.00016**
		GO:0007049	cell cycle	29	2	0.09	6	0.00306	1
		GO:0008150	biological_process	1245	4	4	11	1	1
		GO:0009987	cellular process	809	4	2.6	10	0.17782	1
		GO:0030154	cell differentiation	15	2	0.05	3	0.0008	1
		GO:0032502	developmental process	20	2	0.06	5	0.00144	1
		GO:0048869	cellular developmental process	15	2	0.05	4	0.0008	1
		GO:0050789	regulation of biological process	225	2	0.72	8	0.15168	1
		GO:0050794	regulation of cellular process	206	2	0.66	7	0.12999	1
	CC	**GO:0019028 (E)**	**viral capsid**	**375**	**9**	**4.57**	**1**	**0.0077**	**0.0077**
		GO:0005634	nucleus	49	2	0.6	3	0.1163	0.1163
		GO:0043231	intracellular membrane-bounded organelle	72	2	0.88	4	0.2169	0.2169
		GO:0043227	membrane-bounded organelle	80	2	0.98	6	0.2543	0.2543
		GO:0043229	intracellular organelle	101	2	1.23	7	0.3538	0.3538
		GO:0043226	organelle	110	2	1.34	8	0.3959	0.3959
		GO:0044424	intracellular part	136	2	1.66	9	0.5119	0.5119
		GO:0005622	intracellular	138	2	1.68	10	0.5203	0.5203
		GO:0005623	cell	171	2	2.09	11	0.6477	0.6477
		GO:0044464	cell part	171	2	2.09	12	0.6477	0.6477
ssRNA	MF	**GO:0046789 (E)**	**host cell surface receptor binding**	**31**	**6**	**0.46**	**1**	**2.90×10^{-6}**	**2.90×10^{-6}**
		GO:0005509	calcium ion binding	2	1	0.03	5	0.029	0.029
		GO:0005198	structural molecule activity	327	9	4.85	6	0.034	0.034
		GO:0005200	structural constituent of cytoskeleton	3	1	0.04	7	0.044	0.044
		GO:0004483	mRNA (nucleoside-2'-O-)-methyltransferas...	27	2	0.4	8	0.059	0.059
		GO:0008171	O-methyltransferase activity	27	2	0.4	9	0.059	0.059
		GO:0070008	serine-type exopeptidase activity	27	2	0.4	10	0.059	0.059
		GO:0003725	double-stranded RNA binding	28	2	0.42	11	0.063	0.063
		GO:0003950	NAD+ ADP-ribosyltransferase activity	5	1	0.07	12	0.072	0.072
		GO:0016763	transferase activity transferring pento	5	1	0.07	13	0.072	0.072
	BP	**GO:0006352 (T)**	**DNA-templated transcription initiation**	**4**	**2**	**0.06**	**1**	**0.0013**	**0.0013**
		**GO:0019064 (E)**	**fusion of virus membrane with host plasm...**	**48**	**4**	**0.73**	**3**	**0.0049**	**0.0049**
		GO:0007155	cell adhesion	16	2	0.24	16	0.0233	0.0233
		GO:0006891	intra-Golgi vesicle-mediated transport	3	1	0.05	17	0.0451	0.0451
		GO:0019058	viral life cycle	411	12	6.27	13	0.0064	0.052
		GO:0022610	biological adhesion	27	2	0.41	22	0.0617	0.0617
		GO:0006471	protein ADP-ribosylation	5	1	0.08	24	0.0741	0.0741
		GO:0006486	protein glycosylation	5	1	0.08	25	0.0741	0.0741
		GO:0009100	glycoprotein metabolic process	5	1	0.08	26	0.0741	0.0741
		GO:0009101	glycoprotein biosynthetic process	5	1	0.08	27	0.0741	0.0741
	CC	**GO:0019031 (E)**	**viral envelope**	**164**	**12**	**4.36**	**1**	**0.00032**	**0.00032**
		GO:0009654	photosystem II oxygen evolving complex	1	1	0.03	3	0.02661	0.02661
		GO:0019898	extrinsic component of membrane	1	1	0.03	4	0.02661	0.02661
		GO:1990204	oxidoreductase complex	1	1	0.03	5	0.02661	0.02661
		GO:0043234	protein complex	72	5	1.92	6	0.03611	0.03611
		GO:0033180	proton-transporting V-type ATPase V1 do	2	1	0.05	7	0.05254	0.05254
		GO:0009521	photosystem	3	1	0.08	8	0.0778	0.0778
		GO:0009523	photosystem II	3	1	0.08	9	0.0778	0.0778
		GO:0017119	Golgi transport complex	3	1	0.08	10	0.0778	0.0778
		GO:0044431	Golgi apparatus part	3	1	0.08	11	0.0778	0.0778
